# Prevalence of Anaemia and Iron Deficiency among Primary Schoolchildren in Malaysia

**DOI:** 10.3390/ijerph15112332

**Published:** 2018-10-23

**Authors:** Safii Nik Shanita, Awal Siti Hanisa, Ab Rahim Noor Afifah, Shoo Thien Lee, Kar Hau Chong, Penny George, Abu Bakar Norazida, Siti Balkis Budin, Ilse Khouw, A Karim Norimah, Bee Koon Poh

**Affiliations:** 1 Faculty of Health Sciences, Universiti Kebangsaan Malaysia, 50300 Kuala Lumpur, Malaysia; nikshanita@ukm.edu.my (S.N.S.); hanisa254@gmail.com (A.S.H.); afifahrahim8812@gmail.com (A.R.N.A.); shoothien@hotmail.com (S.T.L.); karhau88@gmail.com (K.H.C.); pennygeorge_ga5p@hotmail.com (P.G.); norazida87@gmail.com (A.B.N.); balkis@ukm.edu.my (S.B.B.); norimahkarim@ukm.edu.my (A.K.N.); 2 FrieslandCampina, 3818 LE Amersfoort, The Netherlands; Ilse.Tan-Khouw@frieslandcampina.com

**Keywords:** anaemia, inflammation, iron deficiency, schoolchildren

## Abstract

The present study aimed to report the prevalence of anaemia and iron deficiency (ID) and to explore the associations among socio-demographic characteristics, nutritional status and inflammation status in the occurrence of anaemia and ID in a nationally representative sample of Malaysian primary schoolchildren. Using data from the South East Asian Nutrition Surveys (SEANUTS), 544 Malaysian children aged 7 to 12 years were included in this secondary analysis. Blood samples were drawn for haemoglobin and serum ferritin analysis while C-reactive protein (CRP) and α-1-acid glycoprotein (AGP) were measured to detect inflammation. Prevalence of anaemia and ID were 4.0% and 5.2%, respectively. There were significantly more anaemic indigenous *bumiputra* children (9.9%) than Chinese children (0.6%). Correction for inflammation did not change the prevalence of ID. More overweight/obese children than thin/normal weight children were found to have elevated acute phase protein (APP). Children with elevated inflammatory markers had significantly higher ferritin level than children without inflammation. Periodic health assessments of anaemia and ID at the population level to monitor and clarify the epidemiology of health problems are required to inform public health policies and strategies.

## 1. Introduction

Globally, around 600 million preschool and school-aged children are affected by anaemia [1]. According to the World Health Organization (WHO) [2], 65.5% of preschool children in Southeast Asia are anaemic. Anaemia is defined by WHO as a condition of insufficient red blood cells to meet physiologic needs [3]. In many studies, the consequences of anaemia in children have been shown to include impaired cognitive performance [4], behavioural problems [5], as well as reproductive disturbances in adolescent girls [6]. Iron deficiency (ID) has been identified as a contributing factor to anaemia. This comes no surprise as iron is the most commonly deficient micronutrient in both developing and industrialized countries [2,3]. However, in countries where thalassemia is a public health problem, these inherited genetic disorder could also result in haemolytic anaemia [7].

Haemoglobin concentration is the most reliable indicator of anaemia at the population level [2] while serum ferritin is often used as an indicator of body iron stores for the diagnosis of ID [3]. While WHO has stated that a low serum ferritin level is an indication of iron deficiency [3], it should be noted that the presence of inflammation can affect serum ferritin concentration. In order to detect the presence of inflammation in seemingly healthy individuals, it is advisable to measure one or more acute-phase proteins (APP) along with the measurement of ferritin [8]; for example C-reactive protein (CRP) and alpha-1-acid glycoprotein (AGP) levels. Information about the presence of inflammation is used to correct serum ferritin concentrations to improve the accuracy of ID diagnosis [9,10].

According to the World Health Organization (WHO), anaemia is a public health problem that needs to be addressed urgently, since almost half of all children below 5 years (43%) globally are affected [2]. In Malaysia, the overall prevalence of anaemia in 2015 for those aged 15 years and above was 24.6% [11]. Among Malaysian preschool and school-aged children, the SEANUTS study reported that prevalence of anaemia and ID was 6.6% and 4.4%, respectively [12]. On the other hand, some studies in several remote locations in Malaysia, have found that the prevalence of anaemia among Malaysian children ranged between 26.2% and 48.5%, while more than half of them reportedly had ID (54.9–70%) [13,14,15]. Besides, it was observed that ID was higher in girls than boys [12]. Low levels of mothers’ education, low household income, sex, nutritional status (specifically stunting and wasting) and parasitic infections were among the factors potentially contributing to anaemia and ID among Malaysian children [13,14,15]. Another possible cause is that children below 5 years old, particularly, may be anaemic because their mothers are anaemic and do not have sufficient iron intake during pregnancy [16]; whereby a study in Malaysia reported 42.3% anaemic pregnant mothers with 51% non-compliant to iron supplementation [17]. Low consumption of micronutrients, especially iron, in the children’s diet may also increase the risk of anaemia and ID [6].

However, the majority of these studies are often small scale and focused primarily on aboriginal and rural populations; and thus, are unlikely to represent the Malaysian children population as a whole [13,14,15]. There is a need for studies that investigate which among the known socio-demographic and nutritional determinants are associated with the occurrence of anaemia and ID at the population level to inform the future development of effective prevention programmes. In particular, the school-aged child population are almost neglected as most studies were carried out among preschoolers and adults. A review of global literature found that, while malnutrition remains a public health issue in school-aged children in developing countries as well as countries in transition, the number of nutrition studies reporting on micronutrient deficiencies among school-going children in the last decade (2002–2009) are scarce and of variable quality, including studies on ID (less than 40 studies) and anaemia (about 80 studies) [18]. Addressing such nutritional deficiencies during the school-age years is particularly significant not only for promoting optimal physical and cognitive development that are centrally important to children’s education and social development, but could also influence (directly or indirectly) workforce economic potential and the health and survival of the future generation due to their potential to persist into adolescence and reproductive years [18,19]. This is in line with the World Health Assembly (WHA) Global Nutrition Targets 2025 on Anaemia, which aim to reduce anaemia prevalence by 50% among women of reproductive age [20].

Thus, the present study aims to determine the prevalence of anaemia and ID and to explore the associations among socio-demographic characteristics, nutritional status and inflammation status in the occurrence of anaemia and ID using a nationally representative dataset of Malaysian primary schoolchildren.

## 2. Materials and Methods

### 2.1. Study Design

A secondary analysis was conducted using the dataset from South East Asian Nutrition Surveys (SEANUTS), which was a multi-disciplinary nutrition survey of 16,744 children conducted in Indonesia, Malaysia, Thailand and Vietnam. This exploratory analysis is to determine the prevalence and factors associated with anaemia and ID among Malaysian primary school-aged children.

SEANUTS Malaysia was a nationally representative cross-sectional study conducted among children aged 6 months to 12 years [12]. Multistage stratified sampling was conducted to recruit children randomly from the major ethnic groups, namely Malay, Chinese, Indian and Others, which included indigenous Sabah *bumiputra*, Sarawak *bumiputra* as well as other *bumiputra*, in all six regions of Malaysia (Northern, Central, Southern, and East Coast regions of Peninsular Malaysia, as well as Sabah and Sarawak).

The present study was conducted according to the guidelines laid down in the Declaration of Helsinki, and all procedures involving human subjects were approved by the Research Ethics Committee of Universiti Kebangsaan Malaysia (Project Code: NN-072-2009). This project was registered in the Dutch Trial Registry as NTR2462. Written informed consent was obtained from the parents or guardians of all participants. Details of the study design and sampling protocol have been described elsewhere [12,21].

### 2.2. Subjects

A total of 872 children aged 3 to 12 years old had their blood drawn for biochemical analyses. Among these children, 254 were younger children aged 4 to 6 years old while 618 were primary schoolchildren aged 7 to 12 years old. Ethics approval for blood collection had been obtained for children aged 2 years and above; however, many parents of younger children were not keen to provide consent for blood withdrawal, leading to a smaller sample size for younger children. Thus, children aged below 7 years were not included in this analysis. A total of 74 out of 618 primary schoolchildren had missing or invalid data on certain blood parameters or socio-demographic variables and were excluded. Hence, only 544 primary schoolchildren, which approximates a third of the SEANUTS Malaysia primary school-age sample, are included in this secondary data analyses (265 boys, 279 girls).

### 2.3. Socio-Demographic and Nutritional Status Variables

Parents or guardians of children provided socio-demographic information, such as age, sex, ethnicity and monthly household income. The children were categorized into four major ethnic groups, namely Malay, Chinese, Indian or Others (mainly indigenous *bumiputra* children). Household income in Malaysian Ringgit (MYR) was categorized into three groups: low (below MYR 2300 per month); middle (between MYR 2300 and MYR 5599 per month); and high (more than or equivalent to MYR 5600 per month) [22]. (Note: USD1 = MYR3.996 as of 22 June 2018).

Anthropometric measurements performed in this study included weight and height measurements. Weight was recorded to the nearest 0.1 kg using a SECA digital scale model 803 (SECA, Hamburg, Germany). Height was measured to the nearest 0.1 cm using a SECA stadiometer model 213 (SECA, Hamburg, Germany). Z-scores for height-for-age (HAZ) and BMI-for-age (BAZ) were determined using WHO AnthroPlus version 1.0.3 (World Health Organization, Geneva, Switzerland). Stunting was defined as HAZ values <−2SD. Thinness was defined as BAZ values <−2SD, whereas overweight and obesity were defined as BAZ values >1SD and >2SD, respectively [23].

### 2.4. Blood Biochemical Assessment

Approximately 6 mL of venous blood was drawn from all subjects by a trained phlebotomist. All the biochemical analyses were conducted by an accredited laboratory (ISO 15189). Haemoglobin was measured by spectrophotometric technique using Sysmex XE5000 (Sysmex, Kobe, Japan) and serum ferritin by immunoassay based on direct chemiluminescence method using ADVIA Centaur (Siemens, Munich, Germany). Anaemia is defined as having hemoglobin level below the cut-off point according to different age range and sex as defined by WHO: (i) <115 g/L for 5 to 11 years; and (ii) <120 g/L for 12 to 14 years [3]. ID was determined based on serum ferritin level as below 15 µg/L for both males and females aged 5 years and older [3]. Children with ID anaemia (IDA) had both ID and anaemia condition. Inflammation markers, CRP and AGP, were measured by using immunoturbidimetry (ADVIA 2400, Siemens, Munich, Germany and Roche, Basel, Switzerland, respectively). Ferritin level was associated with inflammation and therefore it was corrected with inflammatory markers (CRP and AGP) according to different inflammation stages: (i) incubation stage (correction factor: 0.67); (ii) early convalescence stage (correction factor: 0.96); and (iii) late convalescence stage (correction factor: 0.45) [10]. All stages of inflammation were combined into a single group as the number of subjects for each stage was too few for meaningful statistical comparisons.

### 2.5. Statistical Analysis

Data analyses were performed using complex samples techniques in SPSS version 20.0 (IBM Corporation, Armonk, NY, USA), using weight factors based on the Malaysian population census 2010 [24]. Data are expressed as mean and standard error (SE), or percentage with 95% confidence interval (CI), unless otherwise stated. Descriptive statistics are used to describe the socio-demographic, anthropometric and biochemical characteristics and inflammation stages of children. Chi square (χ^2^) statistics were used to test associations between socio-demography and nutritional status with anaemia, ID and inflammation. Complex samples logistic regression analyses with adjusted *p* value using Bonferroni test were performed to assess the differences in percentages within sex, area of residence, income and nutritional status groups. Meanwhile, the differences in percentages within ethnicity groups refer to 95% CI. Mean value differences between inflammation groups and between nutritional status groups were assessed using complex samples general linear model with Bonferroni adjusted *p* value. The significance level was set at *p <* 0.05.

## 3. Results

The socio-demographic characteristics and nutritional status of children are shown in Table 1. The participants comprised 50.1% boys, 62.2% Malays, 80.8% urban dwellers, and 52.6% children from low income families (i.e., monthly household income of less than MYR 2300). Approximately one-third of the children were classified as overweight (14.1%) or obese (18.8%), while only 4.8% were classified as stunted.

Table 2 depicts the haemoglobin and ferritin value based on nutritional status. The overall mean values for haemoglobin, ferritin and corrected ferritin in this study are 132.4 ± 0.5 g/L, 51.0 ± 2.2 µg/L and 46.6 ± 1.6 µg/L, respectively. Among overweight/obese children, the ferritin and corrected ferritin values are significantly higher than thin/normal weight children.

Table 3 presents the prevalence of anaemia and ID by socio-demographic characteristics and nutritional status. Overall, about 4.0% of primary schoolchildren are anaemic, while 5.2% are iron deficient after correcting for inflammation. Only 8.0% of children who are anaemic are also classified as iron deficient (data not shown). The prevalence of anaemia is significantly higher among children of Other (mainly indigenous *bumiputra*) ethnicities (9.9%) than those of Chinese ethnicity (0.6%). Although not statistically significant, children living in rural areas appeared to have higher prevalence of anaemia (5.2% vs. 3.8%) but lower prevalence of ID (1.4% vs. 6.0%) than their urban counterparts. The prevalence of anaemia and ID also tended to be higher among children from low income families (anaemia: 5.8%; ID: 6.4%) and those who were thin/normal weight (anaemia: 5.0%; ID: 6.0%).

Table 4 shows the distribution of children by stages of inflammation. Some 15.8% of children are classified as having certain inflammation or potential infectious diseases, comprising 2.4% in incubation period, and 6.7% in early convalescence, and another 6.7% in late convalescence stage (data not shown). The prevalence of children without inflammation is significantly higher among thin/normal weight group (92.6%) compared to overweight/obese (67.2%) group. Among children who were overweight/obese, the prevalence of inflammation (32.8%) is significantly higher than among thin/normal weight children (7.4%).

As indicated in Table 5, children with inflammation, were observed to have significantly higher ferritin levels than their counterparts who had no inflammation. However, no significant difference was observed for ferritin_corrected_ and haemoglobin levels. Among children without inflammation the prevalence of ID and IDA remained unchanged even after adjustment for inflammatory markers; however, among children with inflammation, prevalence of ID increased from none to 0.4%.

## 4. Discussion

This study reports the nationwide prevalence of anaemia and ID, and the associations among socio-demographic characteristics, nutritional status and inflammation status in the occurrence of anaemia and ID among Malaysian primary schoolchildren. Overall, our findings showed that the prevalence of anaemia and ID were low among primary schoolchildren in Malaysia. The prevalence of anaemia was associated with ethnicity. Indigenous *bumiputra* children were 17 times more likely to be anaemic than Chinese children. Also, the majority of children who were anaemic were from East Coast region of Peninsular Malaysia and Sabah (data not shown). In addition, overweight/obese children were more prone to inflammation compared to their thin/normal-weight cohorts. Children with inflammation, were observed to have significantly higher ferritin level compared to those without inflammation; however, when ferritin level was adjusted for CRP and AGP levels, the difference disappeared and both groups of children had very similar levels of ferritin_corrected_.

The present study found that the prevalence of anaemia was somewhat lower than reported by Poh et al. [12] in the initial analysis of SEANUTS dataset where the prevalence of anaemia was 6.6% among Malaysian children aged 4 to 12 years old. In that previous report, the younger children showed higher prevalence of anaemia than older children (17.6% among rural children aged 4–6 years vs. 5.1% among rural children aged 7–12 years). This trend was similar to another local study of aboriginal children where children aged 1 to 6 years were reported to have higher prevalence of anaemia (36.7%) than those aged 7 to 12 years (25.6%) [13]. The trend is consistent with a report by WHO, which also showed higher prevalence of anaemia among preschoolers (47.4%) than school-going children (25.4%) [25]. Thus, it appears that anaemia is more prevalent among younger children and the lower prevalence of anaemia in our study can be attributed to the fact that younger children were not included in these analyses. Nevertheless, the overall prevalence of anaemia and ID in this present study was also lower than those of primary schoolchildren in other Southeast Asian countries. In Thailand, the prevalence of anaemia among children were 6.6–12.2%, while prevalence of ID was 32.4–37.2% [26]. In Indonesia, 11.7–12.9% of children had anaemia while 1.9–5.3% had ID [27]. Similarly, SEANUTS Vietnam also reported higher prevalence of anaemia (11.4%) and ID (5.6%) in children aged 6 to 12 years old [28].

The prevalence of anaemia was highest, at 9.9%, among children of Other ethnicity, that is, among primarily indigenous *bumiputra* children from Sabah and Sarawak as well as aboriginal (*Orang asli*) children in Peninsular Malaysia. A study by Foo et al. [29] reported that the iron intake among adolescents from a rural community in Sabah was very poor, in which 98% of the adolescents did not meet the Malaysian Recommended Nutrient Intake (RNI) for iron. Similar to other studies, such differences seen in our study may be due to the dietary intake of iron among rural communities in Sabah [29] and aboriginal children in Peninsular Malaysia [14] as well as the differences in food practices among other ethnic groups in East Malaysia [30]. In this study, the prevalence of anaemia and ID was higher among children from low income families and those who were thin/normal weight, although the results were not statistically significant. ID has been associated with the rural area and low household income where another study of rural children in West Peninsular Malaysia also reported similar findings [13]. Although the overall prevalence reported in this study is not high, anaemia has been identified by WHO as a moderate public health problem in Malaysia [2]. Thus, the application of intermittent iron supplement as recommended by WHO as public health intervention to improve iron status and reduce anaemia among children may be necessary, especially among the sub-groups known to have high prevalence of anaemia [1]. As thalassemia is a known contributing factor to anaemia and is prevalent among certain ethnic groups in Malaysia, such as Malay, Chinese, Orang asli in Peninsular Malaysia, and Kadazandusun in Sabah [31,32,33], a screening programme should be prioritised in order to identify carriers of the thalassemia trait [34,35], which will reduce the health burden of the country and may also indirectly reduce prevalence of anaemia.

The present study showed significantly higher prevalence of overweight and obese children with inflammation compared to those who were thin or normal weight. The exact mechanism is still unclear and needs to be further investigated. According to Ferrari et al. [36], chronic inflammation can happen when there is excessive adiposity among European adolescents. They also indicated that CRP values were significantly higher among overweight/obese adolescents than in thin/normal weight adolescents [36]. Obese individuals also have high body fat [37], which means adipocytes are enlarged and could eventually disturb the mechanisms of metabolic homeostasis of body fat mass, which causes macrophages to accumulate and in turn lead to inflammation [38]. Another mechanism could be that excessive intake of fats or other macronutrients without accompanying antioxidant-rich foods or beverages may lead to oxidative stress, which consequently causes inflammatory reaction [39]. Despite the known significant associations between inflammation and obesity, no statistical significance was observed in the prevalence of anaemia and ID between the two groups of body weight status in our study. In fact, the prevalence values appeared to be higher among children who were thin/normal weight than those who were overweight/obese. Thin children are undernourished and may also experience a lack of dietary diversity [40] suggesting that inflammation was not the major contributing factor to iron deficiency. This is in agreement with our results, which show no differences between the corrected prevalence values and those uncorrected for inflammation. The Ferrari et al. study also confirmed that adiposity was sufficient to cause chronic inflammation but was not sufficient to impair iron status and cause iron deficiency [36].

Plasma ferritin is the best marker of ID [3]. As the presence of inflammation is often linked with increased ferritin concentration, CRP and AGP are used to correct the ferritin value. In the present study, the uncorrected ferritin level of children with inflammation was significantly higher compared to their counterparts with no inflammation. This significant finding is likely because iron transport into the blood circulation is inhibited by an iron regulator hormone called hepcidin in response to inflammation [41]. Hence, the body is more dependent on the endogenous iron stores for erythropoiesis rather than dietary iron [10]. Besides, children with inflammation have higher ferritin concentration compared to those without. This result was obtained when referring to ferritin concentration that was unadjusted for inflammation. This finding is similar with results from Knowles et al. [8], where the concentration of ferritin among children with inflammation in Laos was significantly higher compared to children without inflammation before correcting for inflammation. However, after correcting the data for inflammation, ferritin concentrations were lowered in children with inflammation and was similar with ferritin levels in children without inflammation.

The limitation of this study is that the number of children who had their blood drawn was rather small due to lack of parental consent. It was particularly difficult to obtain consent from the parents of younger children leading to the exclusion of preschool-aged children from this secondary analysis. Analysis of subgroups with more than two categories was collapsed into only two groups even though it opposed mechanistic or clinical perspectives. This is due to the small sample size for each subgroup, especially when grouped by various inflammation categories, and merging groups had increased the number of observation for each subgroup and helped reduce the confidence intervals. Nonetheless, the strength of this study is that the sample was weighted to represent the entire population of primary schoolchildren in Malaysia, thus providing a nationally-representative dataset.

## 5. Conclusions

In conclusion, our nationally representative findings showed low prevalence of anaemia and ID among primary school-going children aged 7 to 12 years in Malaysia. Prevalence of anaemia in children was highest among the other ethnicities group, which comprised primarily of Sabah *bumiputras*, Sarawak *bumiputras* and other *bumiputras*, including *Orang asli*. In addition, children who were overweight or obese were more prone to inflammation compared to children who were thin or normal weight. Children with inflammation had higher ferritin levels than those without. Although anaemia and ID in the present study showed an overall low prevalence among Malaysian primary schoolchildren, there were some groups that had higher prevalences. Thus, periodic health surveys are needed to monitor and identify populations at risk, and intermittent iron supplementation may be required as a preventive measure for childhood populations that are at high risk of anaemia and ID.

## Figures and Tables

**Table 1:** Socio-demographic characteristics and nutritional status of children.

	%	Lower CI	Upper CI	Mean	SE
**Age**				9.9	0.1
**Sex**					
Boys	50.1	43.8	56.3		
Girls	49.9	43.7	56.2		
**Ethnicity**					
Malay	62.2	56.5	67.6		
Chinese	20.3	16.4	24.9		
Indian	6.6	4.4	9.6		
Others	10.9	8.2	14.2		
**Area of residence**					
Urban	80.8	76.7	84.3		
Rural	19.2	15.7	23.3		
**Monthly household income ^**‡**^**					
Low (<MYR 2300)	52.6	46.3	58.8		
Medium (MYR 2300–5599)	33.0	27.5	39.0		
High (≥MYR 5600)	14.4	10.7	19.2		
**Anthropometric measurements**					
**Weight (kg)**				34.3	0.7
**Height (cm)**				135.6	0.7
**Body mass index (BMI) (kg/m^2^)**				18.2	0.3
**BMI-for-age (Z-score)**				0.3	0.1
**Height-for-age (Z-score)**				−0.4	0.1
**BMI status**					
Thinness	9.2	5.9	14.0		
Normal weight	57.9	51.6	64.0		
Overweight	14.1	10.2	19.3		
Obese	18.8	14.7	23.7		
**Height**					
Stunted	4.8	2.9	7.7		

Abbreviation: MYR, Malaysian Ringgit; BMI, Body Mass Index. **^**‡**^** USD1 = MYR3.996 (as of 22 June 2018). Cut off point for BMI status: Thinness: −2SD. Overweight: 1SD. Obese: 2SD.

**Table 2:** Haemoglobin and ferritin values according to nutritional status.

	Hb (g/L)		Ferritin (μg/L)		Ferritin_corrected_ (μg/L)	
	Mean	SE	Mean	SE	Mean	SE
**Overall**	132.4	0.5	51.0	2.2	46.6	1.6
**BMI status**						
Thin/normal weight	132.0	0.6	43.8	1.8	42.7	1.7
Overweight/obese	133.2	0.8	65.6 ^a^	4.6	54.5 ^a^	3.2
**Height**						
Stunted	125.1 ^b^	1.4	45.6	5.0	45.3	5.0
Normal height	132.8	0.5	51.3	2.2	46.6	1.7

Abbreviation: Hb, Haemoglobin; BMI, Body Mass Index. ^a^ Significantly different compared to thin/normal weight group at *p <* 0.05 by using General Linear Model analysis. ^b^ Significantly different compared to normal height group at *p <* 0.05 by using General Linear Model analysis. Ferritin_corrected_ is adjusted for CRP and AGP.

**Table 3:** Prevalence of anaemia and ID in children.

	Anaemia		Iron Deficiency		Iron Deficiency_corrected_	
	%	CI	%	CI	%	CI
**Overall**	4.0	2.4–6.7	5.1	2.7–9.4	5.2	2.8–9.4
**Sex**						
Boys	4.5	2.4–8.4	3.1	1.5–6.6	3.1	1.5–6.6
Girls	3.6	1.5–8.1	7.0	3.0–15.5	7.2	3.1–15.6
**Ethnicity**						
Malay	4.2 ^a,b^	2.1–8.4	5.6	2.3–12.8	5.6	2.3–12.8
Chinese	0.6 ^a^	0.1–2.4	2.2	0.5–9.9	2.2	0.5–9.9
Indian	3.3 ^a,b^	0.5–20.4	13.8	6.0–28.8	13.8	6.0–28.8
Others	9.9 ^b^	4.6–19.8	2.2	0.6–7.9	2.8	0.9–8.4
**Area of residence**						
Urban	3.8	2.0–7.0	6.0	3.1–11.3	6.0	3.1–11.3
Rural	5.2	2.5–10.7	1.4	0.6–3.2	1.4	0.6–3.2
**Monthly household income ^**‡**^**						
Low (<MYR 2300)	5.8	3.2–10.5	6.4	2.8–14.0	6.4	2.8–14.0
Medium/High (MYR 2300 and above)	2.0	0.8–5.3	3.6	1.4–8.9	3.8	1.5–8.9
**BMI status**						
Thin/normal weight	5.0	2.8–8.7	6.0	2.8–12.4	6.0	2.8–12.4
Overweight/Obese	2.1	0.6–6.6	3.2	1.4–7.4	3.4	1.5–7.6
**Height**						
Stunted	7.2	1.9–23.1	0	0	0	0
Non-stunted	3.9	2.2–6.6	5.3	2.8–9.8	5.4	2.9–9.9

^a,b^ Different alphabets show that prevalence values are significantly different between socio-demographic and nutritional status variables at *p <* 0.05. Abbreviation: MYR, Malaysian Ringgit; BMI, Body Mass Index. ^**‡**^ USD 1 = MYR 3.996 (as of 22 June 2018). ^**‡**^ Cut off point for BMI status: Thinness: −2SD, Overweight: 1SD, Obese: 2SD. Complex sampling logistic regression was used to analyse the significant differences in percentages within socio-demographic groups and within nutritional status groups; differences in percentages within ethnicity groups referred to 95% confidence interval.

**Table 4:** Distribution of children with or without inflammation by socio-demographic groups.

	Children without Inflammation		Children with Inflammation	
	%	CI	%	CI
**Overall**	84.2	79.2–88.2	15.8	11.8–20.8
**Sex**				
Boys	84.5	78.2–89.3	15.5	10.7–21.8
Girls	83.9	75.5–89.9	16.1	10.1–24.5
**Ethnicity**				
Malay	85.2	77.8–90.5	14.8	9.5–22.2
Chinese	85.2	76.7–91.0	14.8	9.0–23.3
Indian	80.6	58.3–92.5	19.4	7.5–41.7
Others	78.8	64.9–88.2	21.2	11.8–35.1
**Area of residence**				
Urban	85.0	79.0–89.5	15.0	10.5–21.0
Rural	81.2	71.8–87.9	18.8	12.1–28.2
**Monthly household income ^**‡**^**				
Low (<MYR 2300)	82.8	75.1–88.5	17.2	11.5–24.9
Medium/High (MYR 2300 and above)	85.8	78.8–90.8	14.2	9.2–21.2
**BMI status**				
Thin/normal weight	92.6 ^a^	88.2–95.4	7.4 ^a^	4.6–11.8
Overweight/Obese	67.2 ^b^	56.4–76.5	32.8 ^b^	23.5–43.6
**Height**				
Stunted	96.0	84.9–99.0	4.0	1.0–15.1
Non-stunted	83.6	78.4–87.8	16.4	12.2–21.6

^a,b^ Different alphabets show that prevalence values are significantly different between socio-demographic and nutritional status variables at *p <* 0.05. Abbreviation: MYR, Malaysian Ringgit; BMI, Body Mass Index. ^**‡**^ USD 1 = MYR 3.996 (as of 22 June 2018). ^**‡**^ Cut off point for BMI status: Thinness: −2SD, Overweight: 1SD, Obese: 2SD. Without inflammation: neither CRP nor AGP is elevated; With inflammation comprises the following 3 stages–incubation: CRP but not AGP is elevated; early convalescence: both CRP and AGP are elevated; late convalescence: AGP but not CRP is elevated. Complex sampling logistic regression was used to analyse the significant differences in percentage within socio-demographic groups and within nutritional status groups; differences in percentage within ethnicity groups referred to 95% confidence interval.

**Table 5:** Biochemical parameters mean values and prevalence of anaemia, ID and IDA among children with and without inflammation.

	Children without Inflammation			Children with Inflammation		
	Mean	SE	CI	Mean	SE	CI
Hb (g/L)	132.1	0.6	131.1–133.2	133.9	1.1	131.8–136.0
Ferritin (ug/L)	46.8	1.9	43.1–50.5	73.4 *	7.2	59.3–87.5
Ferritin corrected (ug/L)	46.8	1.9	43.1–50.5	45.4	2.6	40.2–50.6
**Prevalence (%)**						
Anaemia	4.5	1.2	2.6–7.6	1.7	1.2	0.4–6.7
ID	6.0	1.9	3.2–11.0	-	-	-
ID corrected	6.0	1.9	3.2–11.0	0.4 *	0.4	0.1–3.1
IDA	0.6	0.4	0.1–2.3	-	-	-
IDA corrected	0.6	0.4	0.1–2.3	-	-	-

Abbreviation: ID, iron deficiency; IDA, iron deficiency anaemia; Hb, haemoglobin. Without inflammation: neither CRP nor AGP is elevated; With inflammation comprises the following 3 stages–incubation: CRP but not AGP is elevated; early convalescence: both CRP and AGP are elevated; late convalescence: AGP but not CRP is elevated. * Significantly different from “without inflammation” group at *p <* 0.05 Complex sampling general linear model was used to analyse the mean differences as compared to without inflammation group; Complex sampling logistic regression is used to analyse the percentage differences as compared to without inflammation group.
